# Genetic structure and mating system of wild cowpea populations in West Africa

**DOI:** 10.1186/1471-2229-12-113

**Published:** 2012-07-24

**Authors:** Eric B Kouam, Remy S Pasquet, Pascal Campagne, Jean-Baptiste Tignegre, Kevin Thoen, Remi Gaudin, Jeremy T Ouedraogo, Abdulai B Salifu, Geoffrey M Muluvi, Paul Gepts

**Affiliations:** 1 International Centre of Insect Physiology and Ecology, P.O box 30772, Nairobi, Kenya; 2 Department of Agriculture, Faculty of Agronomy and Agricultural Sciences, University of Dschang, PO Box 222, Dschang, Cameroon; 3 IRD, Institut de Recherche pour le Développement, UR 072, Laboratoire Evolution, Génomes et Spéciation, UPR 9034, Centre National de la Recherche Scientifique (CNRS), 91198, Gif sur Yvette Cedex, France; 4 Université Paris-Sud 11, 91405, Orsay Cedex, France; 5 CREAF de Kamboinse, INERA, BP 476, Ouagadougou 01, Burkina Faso; 6 Faculté des Sciences, Département de Biologie, BP 243, Niamey, Niger; 7 UMR System 2, place Viala, 34060, Montpellier, France; 8 CSIR-SARI, PO Box 52, Tamale, Ghana; 9 Department of Biochemistry and Biotechnology, Kenyatta University, P.O Box 43844, Nairobi, Kenya; 10 Department of Plant Sciences/MS1, Section of Crop and Ecosystem Sciences, University of California, 1 Shields Avenue, Davis, CA, 95616-8780, USA

## Abstract

**Background:**

Cowpea is a highly inbred crop. It is part of a crop-weed complex, whose origin and dynamics is unknown, which is distributed across the African continent. This study examined outcrossing rates and genetic structures in 35 wild cowpea (*Vigna unguiculata ssp. unguiculata var. spontanea*) populations from West Africa, using 21 isozyme loci, 9 of them showing polymorphism.

**Results:**

Outcrossing rates ranged from 1% to 9.5% (mean 3.4%), which classifies the wild cowpea breeding system as primarily selfing, though rare outcrossing events were detected in each population studied. Furthermore, the analyses of both the genetic structure of populations and the relationships between the wild and domesticated groups suggest possibilities of gene flow that are corroborated by field observations.

**Conclusions:**

As expected in a predominantly inbred breeding system, wild cowpea shows high levels of genetic differentiation and low levels of genetic diversity within populations. Gene flow from domesticated to wild cowpea does occur, although the lack of strong genetic swamping and modified seed morphology in the wild populations suggest that these introgressions should be rare.

## Background

Agricultural systems present spectacular and well studied examples of evolutionary changes [1]. Indeed, crops and their wild relatives represent interesting systems from both agricultural and evolutionary points of view. Wild relatives represent larger amounts of genetic variability than their domesticated descendants and the maintenance of this variability is of central importance in crop conservation and improvement programs [2-4]. Wild relatives may also represent actual or potential weeds and they often constitute crop-weed complexes with the domesticated plants [1,5]. On the other hand, wild relatives are critical for understanding the process of domestication [6-8] as they illustrate how evolutionary forces operate with or without strong artificial selection [9-11].

Cowpea, *Vigna unguiculata* (L.) Walp., plays an important role in the livelihood of millions of poor people in the developping countries of the tropical region where it is used as food, animal feed or as a cash crop [12]. Genetic variability of domesticated cowpea is low [13,14] and most of the genetic variation of this species remains in the wild gene pool [15].

As compared to wild cowpea, domesticated cowpea is characterized by large seeds and non-shattering pods. This crop was domesticated once from its wild progenitor var. *spontanea* somewhere in between Senegal and Eritrea although its precise origin is yet to be established [15]. This domestication took place well before 1500 BC since clearly identifiable domesticated cowpea seeds were found in archaeological deposits dated around 1500 BC, both in central Ghana and in India [16]. Domesticated cowpea experienced a double bottleneck: first from its wild progenitor leading to the primitive cultivar-groups (cv.-gr. Biflora and cv.-gr. Textilis), and then from the primitive cultivar-groups to the evolved cultivar-groups (cv.-gr. Melanophthalmus in West Africa and cv.-gr. Sesquipedalis in Asia) [13]. This partly explains the low diversity of cv.-gr. Melanophthalmus [14].

The wild progenitor of cowpea (*V. unguiculata* subsp. *unguiculata* var. *spontanea* (Schweinf.) Pasquet, formerly known as subsp. *dekindtiana* sensu Verdc.) interbreeds and produces fertile offspring through hybridization with domesticated cowpea (var. *unguiculata*) [17]. This wild progenitor of cowpea is a weed, mostly encountered in cultivated fields and disturbed habitats. This crop-weed complex is distributed over a wide geographical range in Africa [18-20]. However, while the existence of this crop-weed complex is obvious based on both morphological and molecular data, its origin and dynamics remain obscure.

Low levels of gene flow were detected by all authors who have run source and sink trials with cowpea breeding lines: between 0 and 0.85% on average but with some plots where outcrossing rates could reach 4 – 5% [21-23]. Pollinator studies show that pollen may theoretically be dispersed over distances of several kilometers and exchanged between wild and domesticated plants [24]. Nevertheless, the effect of pollen movement on population structure is unknown. Both domesticated cowpea and its wild progenitor are characterized by a flower structure that should promote inbreeding [25]. Accordingly, domesticated cowpea is known as an highly inbred crop [26,27]. Therefore, gene flow between domesticated and wild cowpeas could be negligible.

Last but not least, an insect-resistant genetically engineered (GE) cowpea has been recently developed and may become available to African farmers in the forthcoming years [28]. The GE-cowpea expressing toxins of *Bacillus thuringiensis* targets the pod borer (*Maruca vitrata* Fabricius; Lepidoptera: Pyraloidea: Crambidae), a migrant moth (from Guinean zone where cowpea is a minor crop to Sudan and Sahel zones where cowpea is a major crop) causing almost yearly, often devastating outbreaks for the cowpea crops. This project is currently based on the use of a Cry1Ab gene, although a two genes pyramided construction is expected to be used in the future. The project is currently focusing on Ghana, Burkina Faso, and Nigeria. First confined field trials in Nigeria appear very promising (http://www.aatf-africa.org).

Although autogamous mating systems should favour gene containment in the crop, the possible escape of transgenes encoding insecticidal proteins into wild relative populations might enhance the fitness of wild individuals. In a worst-case scenario, wild cowpea loosing major predators could become a more aggressive weed.

However, wild cowpea has never been studied at the population level and gene flow has never been studied within natural wild cowpea populations. Such studies are necessary to draw a preliminary assessment of the potential fixation and spread of transgenes in natural populations [1].

The present study was undertaken in order to investigate the mating system and the genetic structure of populations in the wild cowpea, in a region encompassing four countries of West Africa, two of them targeted by the *Bt*-cowpea project. We used allozymes as genetic markers to address the following research questions: (1) How inbred are the wild cowpea populations in this region? 2) Is there evidence of gene-flow between populations, especially between domesticated and wild populations of the cowpea gene pool? Both questions appear critical in the light of the forthcoming deployment of GE insect-resistant cowpea in West African countries.

## Results

**Figure 1:**
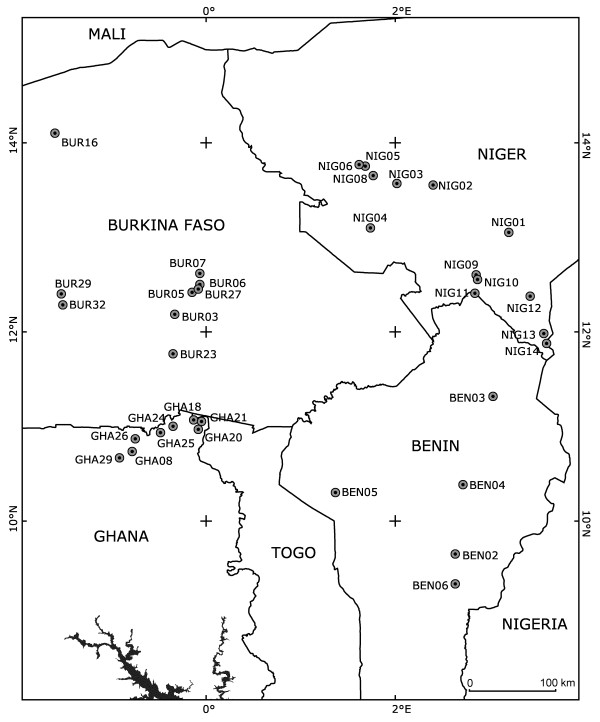
**Geographical locations of the 35 sampling sites reported in Table**1**.** All locations correspond to wild cowpea populations, excepted BEN05, BUR03, GHA26, NIG04, NIG10, NIG12, and NIG13 where wild and domesticated populations were sampled.

A total of 3209 (2977 wild and 232 domesticated) seeds originating from 455 (397 wild and 58 domesticated) plants were analyzed. The genotype of the 397 wild plants was inferred: 52 plants from 8 populations in Ghana, 64 from 9 populations in Burkina Faso, 216 plants from 13 populations in Niger, and 65 plants from 5 populations in Benin (Figure 1 and Table 1).

**Table 1:** Description of the sampling sites: geographic coordinates, habitat and indices of genetic diversity

**Population**	**Latitude**	**Longitude**	**Habitat**	** *N* **	** *P* **	** *Ho* **	** *He* **	** *Fis* **
Wild								
BUR 03	12° 11’ N	00° 20’ W	field	7	0.190	0	0.054	1.000***
BUR 05	12° 25’ N	00° 09’ W	field	6	0.286	0.040	0.061	0.432*
BUR 06	12° 30’ N	00° 04’ W	roadside	5	0.048	0.009	0.009	0.000
BUR 07	12° 37’ N	00° 04’ W	natural	5	0.190	0	0.061	1.000***
BUR 16	14° 06’ N	01° 36’ W	roadside	5	0.143	0.029	0.052	0.538
BUR 23	11° 46’ N	00° 21’ W	roadside	5	0.143	0.009	0.050	0.846**
BUR 27	12° 27’ N	00° 05’ W	natural	12	0.190	0.029	0.077	0.665***
BUR 29	12° 24’ N	01° 32’ W	roadside	11	0.333	0.009	0.103	0.923***
BUR 32	12° 17’ N	01° 31’ W	roadside	8	0.238	0.006	0.092	0.943***
**mean BUR**					0.196 ± 0.028	0.015 ± 0.005	0.062 ± 0.009	0.705 ± 0.112*
GHA 08	10° 44’ N	00° 47’ W	field	7	0.095	0.007	0.006	0
GHA 18	11° 04’ N	00° 08’ W	field	7	0.048	0.020	0.022	0.143
GHA 20	10° 58’ N	00° 05’ W	field	5	0	0	0	m
GHA 21	11° 03’ N	00° 03’ W	field	5	0.095	0.009	0.047	0.833*
GHA 24	11° 00’ N	00° 21’ W	field	5	0.095	0.009	0.035	0.778*
GHA 25	10° 56’ N	00° 29’ W	field	5	0.048	0.009	0.020	0.600
GHA 26	10° 52’ N	00° 45’ W	field	13	0	0	0	m
GHA 29	10° 48’ N	00° 55’ W	field	5	0.048	0.009	0.009	0.000
**mean GHA**					0.054 ± 0.015	0.008 ± 0.002	0.017 ± 0.006	0.392 ± 0.159(*)
NIG 01	13° 03’ N	03° 12’ E	field	6	0	0	0	m
NIG 02	13° 33’ N	02° 24’ E	field	11	0	0	0	m
NIG 03	13° 34’ N	02° 01’ E	field	10	0	0	0	m
NIG 04	13° 06’ N	01° 44’ E	natural/field	37	0.095	0	0.010	1.000***
NIG 05	13° 45’ N	01° 41’ E	roadside	15	0.238	0.035	0.066	0.497***
NIG 06	13° 46’ N	01° 37’ E	roadside	11	0	0	0	m
NIG 08	13° 39’ N	01° 46’ E	roadside	5	0.048	0.019	0.015	- 0.143
NIG 09	12° 36’ N	02° 51’ E	natural	24	0.190	0	0.047	1.000***
NIG 10	12° 33’ N	02° 52’ E	natural	20	0.143	0.005	0.067	0.932***
NIG 11	12° 24’ N	02° 50’ E	natural	18	0.143	0	0.052	1.000***
NIG 12	12° 23’ N	03° 26’ E	natural	18	0.333	0.029	0.094	0.703***
NIG 13	11° 59’ N	03° 34’ E	natural	13	0.238	0.011	0.055	0.806***
NIG 14	11° 53’ N	03° 36’ E	natural	28	0.190	0.011	0.057	0.821***
**mean NIG**					0.124 ± 0.032	0.008 ± 0.003	0.036 ± 0.009	735 ± 0.123(*)
BEN 02	09° 47’ N	02° 38’ E	roadside	5	0.048	0.019	0.015	- 0.143
BEN 03	11° 19’ N	03° 02’ E	natural	8	0	0	0	m
BEN 04	10° 23’ N	02° 43’ E	natural	13	0.048	0.004	0.010	0.647
BEN 05	10° 18’ N	01° 22’ E	field	19	0.095	0.002	0.023	0.898***
BEN 06	09° 20’ N	02° 38’ E	roadside	20	0	0	0	m
**mean BEN**					0.038 ± 0.020	0.008 ± 0.004	0.017 ± 0.005	0.467 ± 0.314 ns
Mean wild					**0.113** ± 0.017	**0.009 ± 0.008**	**0.035 ± 0.020**	**0.657** ± 0.073****
Domesticated								
BUR03	12° 11’ N	00° 20’ W		5	0	0	0	m
BEN05	10° 18’ N	01° 22’ E		6	0	0	0	m
GHA26	10° 52’ N	00° 45’ W		9	0.048	0	0.024	1.000***
NIG 04	13° 06’ N	01° 44’ E		8	0.095	0	0.042	1.000***
NIG 10	12° 33’ N	02° 52’ E		8	0	0	0	m
NIG 12	12° 23’ N	03° 26’ E		12	0.048	0	0.018	1.000***
NIG 13	11° 59’ N	03° 34’ E		10	0.048	0	0.009	1.000***
Mean domesticated					**0.034** ± 0.014	**0** ± 0.000	**0.013 ± 0.006**	**1.000** ± 0.000***

*N*, Number of Individual sampled; *P*, Proportion of polymorphic loci; *Ho*, observed heterozygosity; *He*, Hardy-Weinberg expected heterozygosity; *Fis*, Inbreeding coefficient over the 21 loci. Significant test for departure from Hardy-Weinberg Equilibrium are indicated as *ns* for *P* > 0.1, (*) for *P* < 0.1, * for *P* < 0.050; ** for *P* < 0.010 and *** for *P* < 0.001. *m*, Monomorphic.

Of the 21 loci screened, nine were polymorphic (*Amp2, Amp3, Amp4, Enp, Fdh, Fle3, Pgi2, Pgi3*, and *Pgm1*) with 1.5 alleles per locus at the species level. The *Enp*^*103*^ allele was encountered in a single plant from population BUR05. The *Amp3*^*103*^ and *Enp*^*98*^ alleles were found only in Burkina Faso, while the *Pgm1*^*96*^ and *Pgm1*^*105*^ alleles were found in Niger only. At the population level, 11% of loci were polymorphic and the number of alleles per locus (*A*) was 1.1 (maximum 1.3 in BUR 29 and NIG 12). The effective number of allele *Ae* per population, defined as the inverse of the homozygosity 1/(1-*He*), was 1.032 (±0.005, Standard Error). Eight wild populations – from Benin, Ghana, and Niger – were monomorphic (Table 1).

### Mating system analysis

Wright’s *F*_*IS*_ indicated clear deviations from Hardy-Weinberg equilibrium (Table 1). In the domesticated populations, no heterozygote was found and the overall *F*_*IS*_ estimate was significantly greater than zero (*F*_*IS*_ = 0.657; *P* < 0.001, randomization test) in the wild populations. Fifteen populations were large and polymorphic enough to analyze the mating system of wild cowpea using the MLTR software [29]. The analysis of the mating system was carried out by comparing the inferred genotypes of mother plants to the genotypes of the progenies. High levels of self-fertilization were detected in all populations (Table 2). The average outcrossing rate (*t*) based on multilocus- (*tm*) and single-locus- (*ts*) estimations were significantly lower than 1 (*tm*: *P* < 0.001, *ts*: *P* < 0.001, Wilcoxon signed rank test).

**Table 2:** Outcrossing rates and inbreeding coefficient

**Population**	**Sample sizes**		** *tm* ** **± SE**	** *ts* ** **± SE**	**( *tm-ts* ) ± SE**	** *F* **	** *Fe* **	** *Fe - F* **
	**m**	**n**						
BUR03	112	13	0.014 ± 0.007	0.014 ± 0.007	0.000 ± 0.000	0.649	0.972	0.323
BUR05	105	11	0.095 ± 0.035	0.064 ± 0.024	0.031 ± 0.017	0.458	0.826	0.368
BUR07	148	14	0.061 ± 0.007	0.044 ± 0.006	0.017 ± 0.004	0.990	0.885	−0.105
BUR16	108	13	0.013 ± 0.002	0.014 ± 0.002	0.000 ± 0.000	0.736	0.974	0.238
BUR23	102	11	0.028 ± 0.014	0.020 ± 0.014	0.008 ± 0.003	0.753	0.946	0.193
BUR27	110	12	0.050 ± 0.008	0.038 ± 0.007	0.012 ± 0.003	0.689	0.905	0.216
BUR29	106	12	0.010 ± 0.000	0.010 ± 0.000	0.000 ± 0.000	0.906	0.980	0.074
BUR32	98	11	0.010 ± 0.000	0.010 ± 0.000	0.000 ± 0.000	0.879	0.980	0.101
**Mean BUR**			0.035 ± 0.011**	0.027 ± 0.007**	0.009 ± 0.004(*)	0.758 ± 0.059**	0.934 ± 0.020**	0.176 ± 0.0532*
NIG05	146	15	0.063 ± 0.019	0.020 ± 0.010	0.044 ± 0.014	0.471	0.881	0.410
NIG09	214	24	0.010 ± 0.000	0.010 ± 0.000	0.000 ± 0.000	0.892	0.980	0.088
NIG10	172	20	0.016 ± 0.002	0.012 ± 0.001	0.004 ± 0.001	0.948	0.969	0.021
NIG11	182	18	0.010 ± 0.000	0.010 ± 0.000	0.000 ± 0.000	0.889	0.980	0.091
NIG12	276	28	0.037 ± 0.003	0.025 ± 0.002	0.012 ± 0.001	0.990	0.929	−0.061
NIG13	164	17	0.034 ± 0.004	0.022 ± 0.003	0.012 ± 0.002	0.990	0.934	−0.056
NIG14	126	13	0.058 ± 0.004	0.040 ± 0.003	0.017 ± 0.002	0.990	0.890	−0.100
**mean NIG**			0.033 ± 0.008*	0.020 ± 0.004*	0.013 ± 0.006(*)	0.881 ± 0.070*	0.938 ± 0.016*	0.056 ± 0.065 *ns*
Mean			0.034 ± 0.007***	0.024 ± 0.004***	0.010 ± 0.003**	0.815 ± 0.028***	0.936 ± 0.013***	**0.125** ± 0.043*

*tm* is the multilocus population outcrossing rate, *ts* is the averaged single locus estimate of outcrossing rate, a positive and significant (*tm* – *ts*) reflects biparental inbreeding. *F* is the (minimum variance estimate of) single locus inbreeding coefficient of maternal parents, *Fe* is the expected inbreeding coefficient at equilibrium, (*Fe-F*) quantifies deviation from inbreeding equilibrium based on selfing rate (*Fe*). *tm* and *ts* were tested to differ from 1, the other parameters were tested to differ from zero. Significance levels correspond to the results of non-parametric Wilcoxon signed rank tests, *ns* for *P* > 0.1, (*) for *P* < 0.1, * for *P* < 0.050; ** for *P* <0.010 and *** for *P* < 0.001.

Outcrossing rates estimates and inbreeding coefficient were computed for 15 wild cowpea populations by comparing the inferred maternal genotypes to the progeny genotypes.

In the wild populations, estimates of outcrossing rates respectively based on a multilocus and an averaged single-locus estimator were *tm =* 0.034 ± 0.007 and *ts* = 0.024 ± 0.004, respectively. Outcrossing rate estimates *tm* ranged from 0.01 (BUR29, BUR32, NIG09, NIG11) to 0.095 (BUR05). Therefore, wild *Vigna unguiculata* is a highly inbred plant with up to 97% of self-pollination occurrences (calculated as 1 - *tm*). The amount of biparental inbreeding was assessed with the difference *tm*-*ts*. The average biparental inbreeding (0.010 ± 0.003) differed significantly from zero (*P* = 0.008, Wilcoxon signed rank test) while it represented only 1% of the overall apparent inbreeding, indicating a low but significant occurence of mating between close relatives. In six populations (BUR07, BUR27, NIG05, NIG12, NIG13, NIG14) out of the 15 wild populations, the biparental inbreeding was higher than zero (*P* < 0.05, bootstrap included in the MLTR procedure). Significant heterogeneity in multilocus outcrossing rates (*tm*) was observed among the fifteen populations analysed (*χ²* = 301.81, P < 0.001). This result does not include *tm* values with zero as standard error. We failed to find any correlations between the multilocus outcrossing rate and the population size (Spearman’s *R* = −0.23, *P* = 0.409).

Inbreeding coefficient based on genotypic frequencies of maternal plants (*F*) was positive and significantly greater than zero (Table 2). Expected inbreeding coefficients at equilibrium, as estimated from the multilocus outcrossing rates (*Fe*), were greater than the observed maternal inbreeding coefficients (*F*), a trend that was statistically significant (*P* = 0.026, Wilcoxon signed rank test). The (*Fe* - *F*) values differed significantly (*U*-test, *P* = 0.006) according to the type of habitat (natural vs. disturbed), with a positive median of 0.238 in the disturbed habitats (roadside and field as indicated in Table 1) and a negative median value (−0.018) in less disturbed habitats (i.e., “natural habitats” in Table 1). BEN03, BEN04, BUR07, BUR27 are river and pond banks while NIG10, NIG12, NIG13, NIG14 are partly or totally in “bas-fonds”, partly flooded during the rainy season. Furthermore this difference among habitat types was not related to variations in the *Fe* values (U – test, *P* = 0.517) in these habitats, but was mainly associated with low *F* values (U – test, *P* = 0.012) in disturbed habitats.

### Genetic differentiation and spatial genetic structure

AMOVA (Analysis of Molecular Variance) revealed substantial geographic differentiation in both wild and domesticated populations (Table 3). In the wild populations, a strong and statistically significant genetic differentiation was found at the two hierarchical levels (country and population) indicating a clear geographic structure. Genetic differentiation among populations was high in both wild (*Φ*_*ST*_ = 0.646, *P* < 0.001) and domesticated populations (*Φ*_*ST*_ = 0.574, *P* < 0.001).

**Table 3:** Analysis of Molecular Variance (AMOVA)

**Pool**	**Source**	** *df* **	** *SS* **	** *MS* **	** *σ²* **	**% Total**	**Φ-statistics**
Wild	Among Regions	3	252.232	84.077	0.623	13%	
	Among Populations	32	922.948	28.842	2.522	52%	*Φ*_*RT*_ = 0.128 ***
	Within Populations	360	621.663	1.727	1.727	35%	*Φ*_*SR*_ = 0.594 ***
	Total	395	1796.843		4.872	100%	*Φ*_*ST*_ = 0.646 ***
Domesticated	Among Populations	6	63.138	10.523	1.198	57%	
	Within Populations	50	44.511	0.890	0.890	43%	
	Total	56	107.649		2.088	100%	*Φ*_*ST*_ = 0.574 ***

*df* indicates the degree of freedom, *SS* sum of squares, *MS* mean sum of squares, *σ²* the estimated component of variance attributable to each factor and *Φ* the estimation of the genetic differentiation.. * *P* < 0.050, ** *P* < 0.010; *** *P* < 0.001.

Analysis of Molecular Variance (AMOVA) in both, the wild and the domesticated populations. Two hierarchical levels were analyzed in the wild populations: country level (regions, *R*), population level (*S*) and only one level, the population level (*S*), was analyzed in domesticated populations.

**Figure 2:**
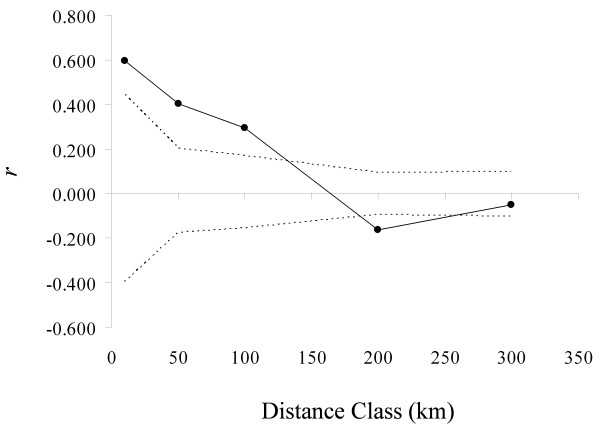
**Pattern of isolation by distance.** Pattern of isolation by distance in the wild populations. Correlogram plot of the genetic autocorrelation coefficient as a function of the geographical distance classes; dotted lines define the 95%-confidence interval based on 999 permutations.

A pattern of isolation by distance was revealed by the regular decay of the coefficient of spatial autocorrelation. Significantly positive autocorrelation was found for the lowest distance classes (up to 100 km), in the wild populations only (Figure 2). A Mantel test did not reveal any significant effect of geographic distance in the domesticated populations (*P* = 0.755, permutations).

### Relationship between wild and domesticated populations

**Figure 3:**
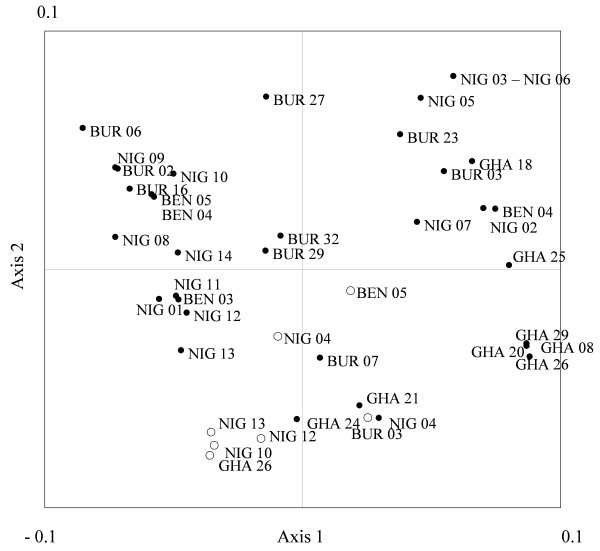
**Principal coordinate analysis.** PCO (Principal coordinate) map (Axes 1 and 2) of the sampled populations based on their genetic distances. White dots correspond to domesticated populations; black dots, wild populations. The two first axes accounted for 73.6% of the total variation (43.2% and 30.4% for axis 1 and axis 2, respectively).

**Figure 4:**
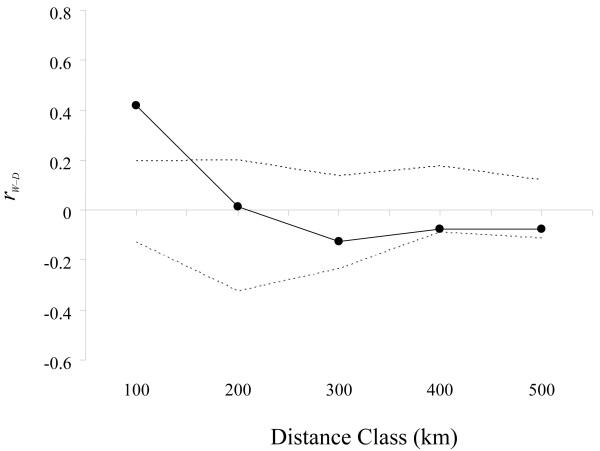
**Autocorrelation analysis.** Autocorrelation analysis of wild-domesticated pairs of populations (*r*_*W-D*_) performed on asymmetrical distance matrices. Plot of the genetic autocorrelation coefficient as a function of the geographical distance classes; dotted lines define the 95%-confidence interval based on 999 permutations.

As expected, domesticated cowpea diversity was very low, without a single heterozygous genotype observed (Table 1, *Ho*). In the Principal Coordinates Analysis (PCO) performed on the inferred genotypes of the wild and domesticated populations (Figure 3), the two first axes accounted for 73.6% of the total variation. Even if some wild populations originating from a same country, such as GHA 08, GHA20, GHA26, GHA29, were associated, no exclusive association based on the country of origin was observed. All domesticated populations were grouped at the negative part of Axis 2. However, four wild populations (BUR07, GHA21, GHA24, and NIG04) were associated with this domesticated group. The last autocorrelation analysis (Figure 4) revealed positive and significant allelic correlations between pairs of wild and domesticated populations at a distance lower than 100 km.

## Discussion

The genetic structure of cowpea populations is highly determined by its mating system characterised by a high selfing rate. Moreover, in line with a previous study [23], our data are also strongly suggestive of genetic exchanges presumably caused by pollinator activity. The genetic variation in *Vigna unguiculata* ssp *unguiculata* var. *spontanea* is low compared to those previously reported in cowpea using allozymes e.g., [15,30,31]. However, these studies surveyed several subspecies and were therefore encompassing a much larger part of the cowpea gene pool. Previous results related to var. *spontanea* (accessions from almost all sub-Saharan Africa) [15] showed higher diversity than the ones reported here, suggesting that West African var. *spontanea* represents just a subset of the diversity of the whole var. *spontanea*. Indeed, the study of Coulibaly et al. [19] based on AFLP markers (Amplified Fragment Length Polymorphism) markers revealed var. *spontanea* to be more diverse in eastern than in western Africa. Population genetic study in a wild cowpea population in East Africa (coastal Kenya) shows much higher outcrossing rates than in West Africa (Kouam, unpublished). Reasons explaining the low genetic diversity in the western populations could therefore include a predominantly selfing mating system and/or loss of genetic diversity occurring after genetic bottlenecks during the colonization of dryer savannas linked to the breeding system change [19].

West African var. *spontanea* could be classified as primarily selfed plant, according to the criterion of Schemske and Lande [32]. In the present study, outcrossing rates *t* ranged from 1 to 9.5% across fifteen populations, with a mean equal to 3.4%. The high rates of apparent selfing in wild cowpea populations are consistent with the cowpea flower morphology [25]. In West African var. *spontanea*, anthers are in contact with the stigmatic surface within the flower bud. Anthers release pollen during the first half of the night [33] and the cuticle that protects the stigmatic surface is ruptured during the second half of the night, which means that pollen can start to germinate on the stigmatic surface a few hours before the opening of the flower (Pasquet, unpublished observations).

Although consistent with previous results, the outcrossing rates we estimated are markedly higher than previous studies based on pollen flow source and sink trials [21-23]. However, the source and sink trials that were used cannot necessarily detect the shortest pollen moves. Our study focused on naturally occurring populations where individuals (eventually both cultivated and wild plants) can stand few cm apart while source and sink trials typically examine pollen flow between spatially clustered groups of plants usually separated by at least one meter.

A low but significant level of biparental inbreeding confirms the local activity of cowpea pollinators in West Africa. Such trends (low outcrossing rates and low biparental inbreeding) are encountered in numerous wild relatives of inbred legume crops [34-40]. Cowpea pollinators either belong to the genus *Xylocopa* or the family Megachilidae (Tignegre, unpublished observations). They visit most of cowpea flowers at least once on average. However, these pollinators are expected to do many more flower-to-flower flights within a flower patch than between flower patches [24,41]. According to Godt and Hamrick [42], the genetic effect of such pollinator behaviour is to reduce the single-locus outcrossing estimates, as observed here. The rather high level of pollinator activity is counteracted by bud self-fertilization (up to 97%). In turn, this mating system leads to almost complete deviations from Hardy-Weinberg equilibrium with a marked heterozygote deficiency [43]. Moreover, the difference between the observed deficit of heterozygotes (*F*) and the theoretical equilibrium based on the estimated selfing rates *tmFe =* (1-*tm*)/(1 + *tm*)], varied according to the ecological context of the populations. In rather undisturbed habitats the observed inbreeding tended to be higher than the inbreeding equilibrium while this trend was reversed in disturbed habitats (field and roadside). Field and roadside are subjected to frequent disturbances and are characterized notably by ground transfers causing possible rearrangements in the soil seed bank. Moreover, outcrossing rates may vary among years [44,45]. This could explain why the expected inbreeding equilibrium derived by selfing rate does not necessarily reflect the parental inbreeding.

Because pollen flow is expected to be sharply reduced when distance increases [24], local gene exchanges should mainly take place within populations, including between wild and domesticated plants when both are mixed or in close proximity. Accordingly, strong genetic differentiation should take place among populations. The existence of spatial genetic structure and its scale of organization is a reflection of gene flow in space and time in relation to the spatial distribution and the colonization history of populations. A spatial genetic structure was found for proximate wild cowpea populations up to 100 km, which reflects a decreased probability to observe related individuals as the distances between populations increase. This suggests genetic exchanges among populations; however, our results do not directly shed light on the patterns of occurrence of gene flow in space and time. Gene flow via pollen in cowpea is likely to occur up to few km with a very low probability of long distance pollen dispersal, and, in any cases, pollen movement is unlikely at distances over 10 km [24]. On the other hand, seed flow through ingestion by grazing mammals could involve much longer distances, though the percentage of seed survival through grazing mammal gut does not exceed a few per cent (Pasquet, unpublished observations). Wild cowpea is expected to express high levels of genetic differentiation and low levels of within-population genetic diversity. In this study, high genetic differentiation was observed at several spatial levels.

Significant allelic correlations between wild and domesticated pairs located in a same zone (distance < 100 km) suggested possibilities of genetic exchanges between these two compartments. Such correlations could arise from multiple local domestications of cowpea. However, considering that cowpea domestication took place more than 3500 years (or generations) ago, and that cv.-gr. Melanophthalmus is the result of two bottlenecks which are likely to have occurred in different places in Africa [13], an alternative explanation for the allelic correlations must be proposed and is supported by results from Pasquet [15], Coulibaly [19], and Feleke [20], respectively based on allele *Amp2*^*102*^, AFLP alleles, and a chloroplastic DNA. In these studies, alleles characterizing domesticated cowpea accessions, which are rather unfrequent in wild cowpea accessions, could have been useful to locate the center of origin of the crop. Surprisingly, such alleles were found to be widespread across Africa. The authors concluded that the presence of these alleles in wild cowpea accessions was the result of introgression of domesticated alleles into wild gene pool. Our present results represent a more direct evidence of these introgressions into wild cowpea populations.

In an area largely dominated by the cultivation of cv. gr. Melanophthalmus which is characterized by three recessive traits, i.e., white colored seeds, thin and wrinkled seed testa, as well as non-shattering pods [46], gene flow between wild and domesticated cowpea is expected to be highly asymmetrical [20]. Because the probability of wild dominant alleles entering the domesticated gene pool is almost null, the asymmetry is expected to be higher in cowpea than in other crops where the phenomenon has been observed [39,47-52]. If a farmer sows a seed from a domesticated flower that has been fertilized by wild pollen, the F_1_ plant will show shattering pods and smaller seeds with a thick and dark testa. Farmers are not likely to select such small seeds with thick and dark testa for the next sowing, and therefore prevent the introduction of wild alleles into the domesticated genepool in areas where cv.-gr. Melanophthalmus is cultivated exclusively. Of course, pollen from such an F_1_ plant may fertilize a flower from a domesticated plant, but the probability of recovering the domesticated phenotypes (i.e., finding the combination of recessive alleles in the progeny of such a natural BC plant) is very low in the end. This hypothesis is confirmed for domesticated cowpea by the near-absence of variability in its populations as well as the absence of some of the alleles encountered in wild cowpea (*Amp4*^*91*^ and especially *Amp2*^*100*^).

Because wild and domesticated plants still co-exist, it is likely that positive and negative factors affecting the survival of hybrids balance each other out. Genetic swamping by domesticated genes would lead to the disappearance of wild types, which is obviously not the case. With the exception of one single wild plant, no large-seeded wild cowpea was collected. The exception produces partly white seeds and resulted probably from introgression with domesticated genes. However, this situation appeared to be an exception to a general rule. If gene flow from domesticated to wild cowpea does exist, the lack of strong genetic swamping and modified seed morphology in the wild populations suggests that these introgressions should be rare. Alternatively, gene flow might be rather frequent while hybrids in non-cultivated environment are expected to be less fit. The white seed color makes seeds more visible to seed predators, the thinner seed coat makes seed less dormant, and reduced seed shattering could reduce dispersal distances.

## Conclusions

Our results showed high selfing rates in the wild cowpea populations, nevertheless possibilities of genetic exchanges within and between West African cowpea populations do exist. Numerous wild or weedy populations grow within cultivated fields or in field margins [18], well within the distance over which cowpea pollen can be transported [22-24]. Regarding the introduction of GE insect-resistant cowpea in West Africa, the escape of the transgene into the wild gene pool will just be a matter of time, even if it is likely that the move will be slow.

There may be technologies to mitigate or prevent gene flow in the future but, in the mean time, the focus should be on determining whether there are any fitness gains provided by a transgene inserted into the genome of a cowpea wild relative [53,54]. If these fitness gains are negligible, there would not be any major problem associated with gene escape accompanying deployment of insect-resistant cowpea in West Africa. Conversely, a cowpea wild relative could become a more troublesome weed in fields and other disturbed areas, given a potential fitness gain provided by an insect resistance transgene. Therefore, an assessment of the potential fitness benefits of transgenes is a prerequisite for the deployment of GE insect resistant cowpea in West Africa.

## Methods

### Plant material

Wild cowpea (*Vigna unguiculata ssp. unguiculata var. spontanea*) seeds were sampled in 35 populations from West Africa (Table 1, Figure 1). The eighteen populations from Niger and Benin were sampled in September-October 1995, the eight populations from Ghana in September-October 2002, and the nine populations from Burkina Faso in October 2003. Some domesticated cowpeas were sampled as well (Table 1). With the exception of the area where BEN05 was collected, the cultivar-group Melanophthalmus was exclusively cultivated in the whole sampling area. The low genetic diversity of this cultivated group [13,14] did not justify an intensive sampling.

Most of these populations were collected in disturbed areas (fields, field margins, recent fallows, and roadsides). Few places looked undisturbed but the lack of disturbance could also be the outcome of a prolonged fallow period. Searches for a truly natural population were made in the ‘Parc du W’ in Niger but not a single wild cowpea population was found there while large populations were found not far from the park boundaries but in cultivated, and therefore disturbed areas. Three categories of habitats could be distinguished: fields, roadsides (both are disturbed habitats) and seemingly undisturbed habitats (denoted “natural” in Table 1). Seeds collected had sizes within the range expected for wild cowpea (10–46 mg with an average of 29 mg) with the exception of one plant from the population BUR05. This plant produced much larger seeds (115 mg); a small part of the testa was white-colored, which suggested that it was the progeny of a wild-domesticated hybrid.

In total, 5 to 37 individual plants were sampled per population. One to three pods per plant were collected and kept separately. Pods were collected from a single inflorescence peduncle or from two adjacent peduncles, in order to be sure that the pods were originating from the same plant. To reduce the probability of duplicate sampling, sampled individuals were separated by a minimum of 10 m. This precaution led in some instances to a low number of plants analyzed. Populations collected in cultivated fields, especially in Ghana, were often reduced to a very few wild plants remaining in the field after several weeding operations. Seeds were stored at -20^o^C until the laboratory analysis was carried out.

Four to twelve seeds per pod were analyzed with isozymes using horizontal starch gel electrophoresis, with a total of 3209 (2977 wild and 232 domesticated) seeds from 479 (421 wild and 58 domesticated) pods analyzed.

### Electrophoresis analysis

Ten enzyme systems revealing 21 putative loci were screened: aminopeptidase (AMP, E.C. 3.4.11.1), endopeptidase (ENP, E.C. 3.4-.-), fluorescent esterase (FLE, E.C. 3.1.1.-), formate dehydrogenase (FDH, E.C. 1.2.1.2), isocitrate dehydrogenase (IDH, E.C. 1.1.1.42), malate dehydrogenase (MDH, E.C. 1.1.1.37), phosphoglucomutase (PGM, E.C. 5.4.2.2), phosphogluconate dehydrogenase (PGD, E.C. 1.1.1.43), phosphoglucose isomerase (PGI, E.C. 5.3.1.9), and shikimate dehydrogenase (SDH, E.C. 1.1.1.25). The targeted enzyme systems were expected to show polymorphism within West African var. *spontanea*[15]. In addition, they were known to be clearly expressed in seeds, either from the wild or from greenhouse, and no null allele has ever been recorded within *V. unguiculata* subsp. *unguiculata* with these enzyme systems.

Seeds were soaked in deionised water overnight to initiate germination prior to enzyme expression. Once dehulled, seeds (germ and cotyledons) were crushed with distilled deionised water using a porcelain mortar and pestle. Enzyme extracts were adsorbed onto 3 mm Whatman filter paper wicks and applied to a 14% starch gel [55]. All enzyme systems were assayed in citrate/histidine buffer system (pH 6.0) with the electrode buffer consisting of 0.41 M citric acid trisodium salt, pH 6.0 and gel buffer comprising 5 mM L-histidine mono HCl 2.5 mM NaCl, pH 6.0. Electrophoresis was carried out at 200 V at 4°C for about three hours. Enzyme-specific staining was carried out according to Wendel and Weeden [56] using either leucine-b-naphtylamide or alanine-b-naphtylamide for AMP, and 4-methyl-umbelliferyl acetate for FLE.

For each enzyme system, we numbered as “1” the presumed locus encoding the most anodally migrating bands; additional loci were numbered sequentially with decreasing electrophoretic mobility. The most common allele was designated by 100 for each locus and others were measured in millimetres of increased or decreased mobility in relation to this standard, using the same nomenclature as in Pasquet [15].

### Data analysis

The genotype of each mother plant was first inferred from the progeny array following the Brown and Allard [57] method and using the MLTR computer program, version 2.2 [29]. The analysis of the mating system was carried out by comparing inferred mother plants genotypes to the genotypes of the progenies. Subsequent analyses were based on the mother plant genotypes only.

#### Mating system

First, the outcrossing rates *t* were estimated in the wild populations expressing three or more polymorphic loci using the MLTR computer program [29], an extension of the original program of Ritland and Jain [58] based on a mixture of outcrossing and self-fertilization events. This procedure estimates: (i) a multilocus outcrossing rate (*tm*); (ii) a single locus estimate of outcrossing rate (*ts*) averaged across loci;(iii) the crossing between relatives and the average maternal plant inbreeding coefficient based on progeny genotypes *F* = the (minimum variance) single locus inbreeding coefficient of maternal parents]. Standard errors of the mating system parameters were estimated based on 500 bootstraps considering the maternal family array as the resampling unit.

*F* was then compared to the expected inbreeding coefficient at equilibrium *Fe* = (1-*t*)/(1 + *t*) [59]. If populations are at a genetic equilibrium and genotypic frequencies are determined solely by the mating system, *F* and *Fe* are equal [60]. Discrepancies between *F* and *Fe* are expected to reflect the amount by which a population deviates from inbreeding equilibrium. *FeF* was tested for difference from zero using a paired non-parametric Wilcoxon signed rank test. Moreover, any differences in (*Fe* – *F*) values according to the degree of disturbance in the habitat of collection, i.e., natural vs. disturbed (i.e., roadside and field), were checked using a Mann – Withney *U* test.

Lastly, in order to verify the contribution of biparental inbreeding versus autogamy to observed inbreeding, we compared *tmts* to zero using a Wilcoxon signed rank test. A *tmts* > 0 value means that biparental inbreeding does exist beside selfing of flowers. To test the heterogeneity of *tm* among the populations analysed, a *Chi-square* test was carried out, as suggested by Godt and Hamrick [42]. The test was carried out by subtracting each population estimate from the global average, dividing these differences by the standard error associated with the outcrossing rate of each population, squaring these quantities, and summing over populations. Under the hypothesis of homogeneity, this statistic was assumed to be *Chi-square* distributed, with *n* – 1 degrees of freedom where *n* is the number of populations. At the population level, diversity parameters were estimated using the Popgene software version 1.3 [61]. These included allele frequencies, percentage of polymorphic loci (*P*), observed heterozygosity (*Ho*), and the expected heterozygosity under Hardy-Weinberg equilibrium (*He*). Fixation indices were estimated with Fstat, version 1.2 [62]. Inbreeding coefficient (*F*_*IS*_) were computed to assess the deviation from Hardy-Weinberg equilibrium following Weir and Cockerham [63] and were tested using a randomization test.

#### Genetic differentiation and spatial genetic structure

Genetic structure among populations was studied by analyzing both genetic differentiation and spatial genetic autocorrelation in the wild and domesticated populations.

First, an analysis of molecular variance (AMOVA) [64] was carried out to test the effects of the following hierarchical levels: country of origin, population. Because of the low number of populations sampled in domesticated cowpea, the full hierarchical analysis was performed only for wild populations. The AMOVA was performed with R software, libraries ape and pegas [65], using pairwise genetic distances following Smouse and Peakall [66]. These different components were tested by Monte-Carlo permutations (n = 999).

Second, spatial autocorrelation of the wild populations was analyzed using the GenAlEx 6.1 software program [67]. Genetic distances for each pairwise combination of populations were estimated according to Nei [68]. In the case of wild cowpea populations, the resulting matrix was used to compute the autocorrelation coefficient *r*[66]. *r* values were tested with 999 permutations. In the case of domesticated cowpea, represented by 7 populations only, spatial autocorrelation was assessed with a Mantel’s test (999 permutations) between Nei’s distances and geographic distances.

#### Relationship between wild and domesticated populations

The possibility of genetic exchange between the wild and domesticated compartment were studied as follows.

A Principal Coordinates Analysis (PCO) ([65], library ade4) was performed on Nei’s genetic distances.

Based on the algorithm published in Smouse and Peakall [66], the computation of the spatial autocorrelation coefficient *r*_*W-D*_ between wild (W) and domesticated (D) populations was implemented in the R software [65] in order to deal with two paired asymmetrical matrices of distance Wild (rows) × Domesticated (columns). Such matrices reported spatial and genetic distances between couples of Wild – Domesticated (W-D) populations only. The spatial allelic autocorrelation coefficient (*r*_*W-D*_) for Wild - Domesticated populations pairs was computed for several distance classes (100 km up to 500 km) and tested with 999 permutations.

## Authors’ contributions

RSP, JBT, KT, RG, JTO and ABS contributed to plant material collection. EBK performed lab analyses. EBK, RSP and PC conceived of the study, analyzed data, interpreted results and wrote the paper. GMM and PG reviewed the paper prior to submission and provided valuable comments on the interpretation and presentation of results. RSP secured funding. All authors read and approved the final manuscript.
